# Comparative genomics of *Vibrio toranzoniae* strains

**DOI:** 10.1007/s10123-024-00557-z

**Published:** 2024-07-12

**Authors:** Rubén Barcia-Cruz, Sabela Balboa, Alberto Lema, Jesús L. Romalde

**Affiliations:** 1 Departamento de Microbiología y Parasitología, CIBUS-Facultad de Biología, Universidade de Santiago de Compostela, Campus Vida S/N, 15782 Santiago de Compostela, Spain; 2 Centro de Investigación Interdisciplinar en Tecnología Ambientales (CRETUS), Universidade de Santiago de Compostela, 15782 Santiago de Compostela, Spain; 3 Present Address: French Agency for Food, Environmental and Occupational Health and Safety (Anses), 94701 Maisons-Alfort Cedex, France; 4 Present Address: AllGenetics & Biology SL, Oleiros, 15172 Perillo, A Coruña Spain

**Keywords:** *Vibrio toranzoniae*, Genome sequencing, Phylogenomics, Virulence genes

## Abstract

*Vibrio toranzoniae* is a marine bacterium belonging to the Splendidus clade that was originally isolated from healthy clams in Galicia (NW Spain). Its isolation from different hosts and seawater indicated two lifestyles and wide geographical distribution. The aim of the present study was to determine the differences at the genomic level among six strains (4 isolated from clam and 2 from seawater) and to determine their phylogeny. For this purpose, whole genomes of the six strains were sequenced by different technologies including Illumina and PacBio, and the resulting sequences were corrected. Genomes were annotated and compared using different online tools. Furthermore, the study of core- and pan-genomes were examined, and the phylogeny was inferred. The content of the core genome ranged from 2953 to 2766 genes and that of the pangenome ranged from 6278 to 6132, depending on the tool used. Although the strains shared certain homology, with DDH values ranging from 77.10 to 82.30 and values of OrthoANI values higher than 97%, some differences were found related to motility, capsule synthesis, iron acquisition systems or mobile genetic elements. Phylogenetic analysis of the core genome did not reveal a differentiation of the strains according to their lifestyle (commensal or free-living), but that of the pangenome indicated certain geographical isolation in the same growing area. This study led to the reclassification of some isolates formerly described as *V. toranzoniae* and demonstrated the importance of cured deposited sequences to proper phylogenetic assignment.

## Introduction

*Vibrio toranzoniae* is a marine bacterium of the Splendidus clade that belongs to the *Vibrio* genus (Lasa et al. 2013). To date, the Splendidus clade is the largest clade within the genus *Vibrio*, containing 18 species: *V. artabrorum, V. atlanticus*, *V. celticus*, *V. chagasii*, *V. coralliirubri*, *V. crassostreae*, *V. cyclitrophicus*, *V. echinoideorum, V. fortis*, *V. gallaecicus*, *V. gigantis*, *V. kanaloae*, *V. lentus*, *V. pelagius*, *V. pomeroyi*, *V. splendidus*, *V. tasmaniensis*, and *V. toranzoniae* (Pérez-Cataluña et al. 2016; Poli et al., 2018; Hira et al. 2019; Jiang et al. 2022). Another species, ‘*V. profundi*’ has been described as belonging to this clade, but has not yet been validated (Zhang et al. 2019). The Spendidus clade comprises several pathogenic species, such as *V. crassostreae* (Bruto et al. 2017), *V. tasmaniensis* (Duperthuy et al. 2011; Rubio et al. 2019), and *V. splendidus* (Thomson et al. 2005), which can cause considerable losses in the aquaculture industry (Dubert et al. 2017). Several virulence factors have described in *Vibrio* species pathogenic for poikilotherm animals, including capsular polysaccharides, adhesive factors, cytotoxins, lipopolysaccharides, and flagella (Amaro et al. 2020).

*Vibrio toranzoniae* was first isolated from clams (*Ruditapes phillipinarum* and *R. decussatus*), during a study of the microbiota associated with reared healthy clams in Galicia, Spain (Lasa et al. 2013). The subsequent isolation of the species from seawater in Valencia (Spain) and from seawater and hatchery rearing systems for the production of blue mussels (*Mytilus galloprovincialis*) in Australia (Kwan and Bolch 2015) indicated that the host and geographical distribution of this species were wider than expected. Additionally, three isolates from moribund red conger eel (*Genypterus chilensis*) in Chile were initially attributed to *V. toranzoniae*, although in this work these isolates were reclassified as *V. kanaloae*.

The genomes of bacterial species provide essential information for elucidating the taxonomy of closely related species and identifying properties of interest, such as drug sensitivity or virulence factors. To address such information, the genomics revolution that made thousands of prokaryotic genomes available to the scientific community has come hand in hand with a revolution in computational tools to compare these genomes (Setubal et al. 2018). The development of bioinformatics tools and web-based databases provides an online, user-friendly method for identifying and predicting relevant information from genomic data, such as antimicrobial resistance information (Babiker et al. 2019) or virulence factor gene information (Waseem et al. 2017). The resulting differences between the genomes of interest may be crucial for deciphering the genetic basis of pathogenicity or virulence capacities among strains, as such information is highly relevant for tracing mortality outbreaks. In addition, some clues about the different survival strategies that vibrios can develop to persist in the environment may be provided.

In this work, we used general genomic features, variable characteristics in factors of interest, evidence of genomic exchange, phylogenetic relationships, and the study of the core- and pan-genomes to compare a collection of *V. toranzoniae* strains. We provide an example of how comparative genomics can help to unravel the taxonomy of a complicated group and how it can help to obtain information regarding the biology of the group.

## Materials and methods

### Bacterial strains

Strains included in the comparison analysis are listed in Table 1. These strains included four motile, facultative anaerobic marine strains isolated from healthy cultured adult clams (*R. philippinarum* and *R. decussatus*) in Galicia (Spain), including the type strain of the species Vb 10.8^ T^ (= CECT 7225^ T^), and two environmental strains isolated from seawater in Valencia (Spain) (kindly donated by Prof. M.J. Pujalte). Additionally, the three strains isolated from red conger eel (*Genypterus chilensis*) in Chile (Lasa et al. 2015), were initially added to the comparisons, until the study revealed that they belong to *V. kanaloae* species. Stock cultures of the isolates were stored at − 80 °C in marine broth supplemented with 20% (v/v) glycerol, and routinely cultured on marine agar plates at 25 °C.

**Table 1:** *Vibrio toranzoniae* strains included in the study

Strain	Origin	Host	Date
Vb 10.8^ T^	Galicia, Spain	*Ruditapes decussatus*	2004
CMJ 9.4	Galicia, Spain	*Ruditapes philippinarum*	2005
CMJ 9.11	Galicia, Spain	*Ruditapes decussatus*	2005
Cmf 13.9	Galicia, Spain	*Ruditapes philippinarum*	2005
96–373	Valencia, Spain	Seawater	1996
96–376	Valencia, Spain	Seawater	1996

### Genomic DNA extraction, sequencing, assembly, and annotation

Genomic DNA was extracted using the QIAamp DNA Mini Kit (Qiagen), following the manufacturer’s protocol. The genomes of *V. toranzoniae* strains were sequenced at David H. Murdock Research Institute (DHMRI) of the University of North Carolina (Kannapolis, North Carolina) using HiSeq 2500 sequencing technology (Illumina) with 2 × 100-bp paired-end reads, and at FISABIO (Valencia, Spain) using a MiSeq system sequencing technology (Illumina) with 2 × 300-bp paired-end reads. Additionally, the genomes of the type strain Vb 10.8^ T^ and the environmental isolate 96–376 were sequenced at SNPsaurus at the University of Oregon, using a PacBio technology.

The Illumina reads were analyzed for quality control using FASTQC (Brabaham Bioinformatics). The reads were trimmed and filtered to remove adapters and low-quality bases, using Trimmomatic 0.32 (Bolger et al. 2014) program. The remaining reads were subjected to genome assembly via the SPAdes 3.6.1, the novo assembler tool (Nurk et al. 2013), and QUAST (Gurevich et al. 2013) software was used to evaluate the assembly.

The whole genomes of the strains were deposited in GenBank under the accession numbers GCA-001541335.1 (*V. toranzoniae* Vb 10.8^ T^), GCA-009906155.1 (*V. toranzoniae* 96–373), GCA-009906235.1 (*V. toranzoniae* 96–376), GCA-009906185.1 (*V. toranzoniae* CMJ 9.4), GCA-009906175.1 (*V. toranzoniae* CMJ 9.11), and GCA-009906085.1 (*V. toranzoniae* Cmf 13.9).

### Genomic indices

To measure the similarity among the strains, in silico DNA-DNA hybridization (dDDH) and the Orthologous Average Nucleotide Identity (OrthoANI) were calculated between pairs of genomes. dDDH was calculated with GGDC software, using the results offered by formula 2 (Meier-Kolthoff et al. 2013). OrthoANI was calculated using ChunLab’s Orthologous Average Nucleotide Identity Tool (OAT), with an algorithm demarcation cutoff of 95 ~ 96% (Lee et al. 2016).

### Sequence correction

Obtention of long sequencing reads has been associated with low sequencing accuracy. Thus, several approaches, such as hybrid assemblies, higher sequencing coverage, or sequence correction, have been proposed to increase the quality of long sequence reads (Mahmoud et al., 2019). In this work, complementation of PacBio low-accuracy long reads with Illumina high-accuracy short reads was performed for both Vb 10.8^ T^ and 96–376 strains. Therefore, the PacBio sequenced genomes were first assembled with Flye version 2.6 (Kolmogorov et al. 2019). Next, Minimap2 version 2.17 (Li 2018) was used to map the genomes back. Then, PacBio sequences were polished with Racon version 1.4.3 (Vaser et al. 2017). After that, alignment with Illumina sequences was achieved with Bowtie2 version 2.3.5 (Langmead and Salzberg 2012). Finally, the results were polished with and Pilon version 1.2.3 (Walker et al. 2014) to construct the hybrid genome.

### Differential phenotypical features

The exploration of genes and systems within the strains was accomplished using different annotation tools, the Rapid Annotations using Subsystems Technology (RAST) server (Overbeek et al. 2014), the Annotation Tools of PATRIC 3.5.43 server (Brettin et al. 2015), and PROKKA V1.13.3 (Seemann 2014).

To corroborate the results observed in the genomic analyses, several biochemical tests were carried out. Capsule production was assessed by culturing the *V. toranzoniae* strains on Congo red agar (CRA) plates as described by Freeman et al. (Freeman et al. 1989). After incubation for 48 h at 25 °C, the black colonies were considered as capsule producers. Detection of siderophores was assayed by culturing the strains on chrome azurol S (CAS) blue agar plates, with orange halos around the colonies indicating siderophore production (Schwyn and Neilands 1987; Lynne et al. 2011). Finally, motility was observed via optical microscopy and soft agar. The presence of flagella was determined by specific staining using Leifson dye (Leifson 1930), and visualizing the preparations were visualized under a 100 × optical microscope.

### Genomic exchange

Different online tools were used to search for genetic transfer. Therefore, antiSMASH 5.0 (Blin et al. 2019) was utilized for identifying secondary metabolite clusters; PHASTER (Arndt et al. 2016), to identify prophages sequences; DefenseFinder (Abby et al. 2014; Tesson et al. 2022), to detect known anti-phage systems; and Comprehensive Antibiotic Resistance Database (CARD) (Alcock et al. 2019), for the detection of antimicrobial resistance genes, using the resistance gene identifier (RFI) tool. Identification of genomic islands was performed with IslandViewer 4 (Bertelli et al. 2017) using IslandPick, SIGI-HMM. and IslandPath-DIMOB methods employing *V. splendidus* LGP32, *V. vulnificus* YJ016, and *V. anguillarum* 775 as the reference genomes. To search for CRISPR-Cas sequences, the genomes were analyzed using CRISPRCasFinder online tool (Couvin et al. 2018).

### Phylogenetic analysis

Core and pangenome phylogenomic analyses of the species were performed using the three different algorithms of GET_Homologues software (Contreras-Moreira and Vinuesa 2013), namely, bi-directional best-hits (BDBH), Cluster of Orhologous Groups triangle (COGtriangle), and Markov Clustering of Orthologous (OrthoMCL). For the appropriate use of GET_Homologues, functional annotation of the genomes was carried out with PROKKA V1.13.3.

Core and pangenome analyses were also performed using Roary software (Page et al. 2015). Phylogenomic trees were visualized using FigTree version 1.4.3 (Rambaut 2016).

## Results and discussion

### Reclassification of the former *V. toranzoniae* R17 strain as *V. kanaloae* R17

**Fig. 1:**
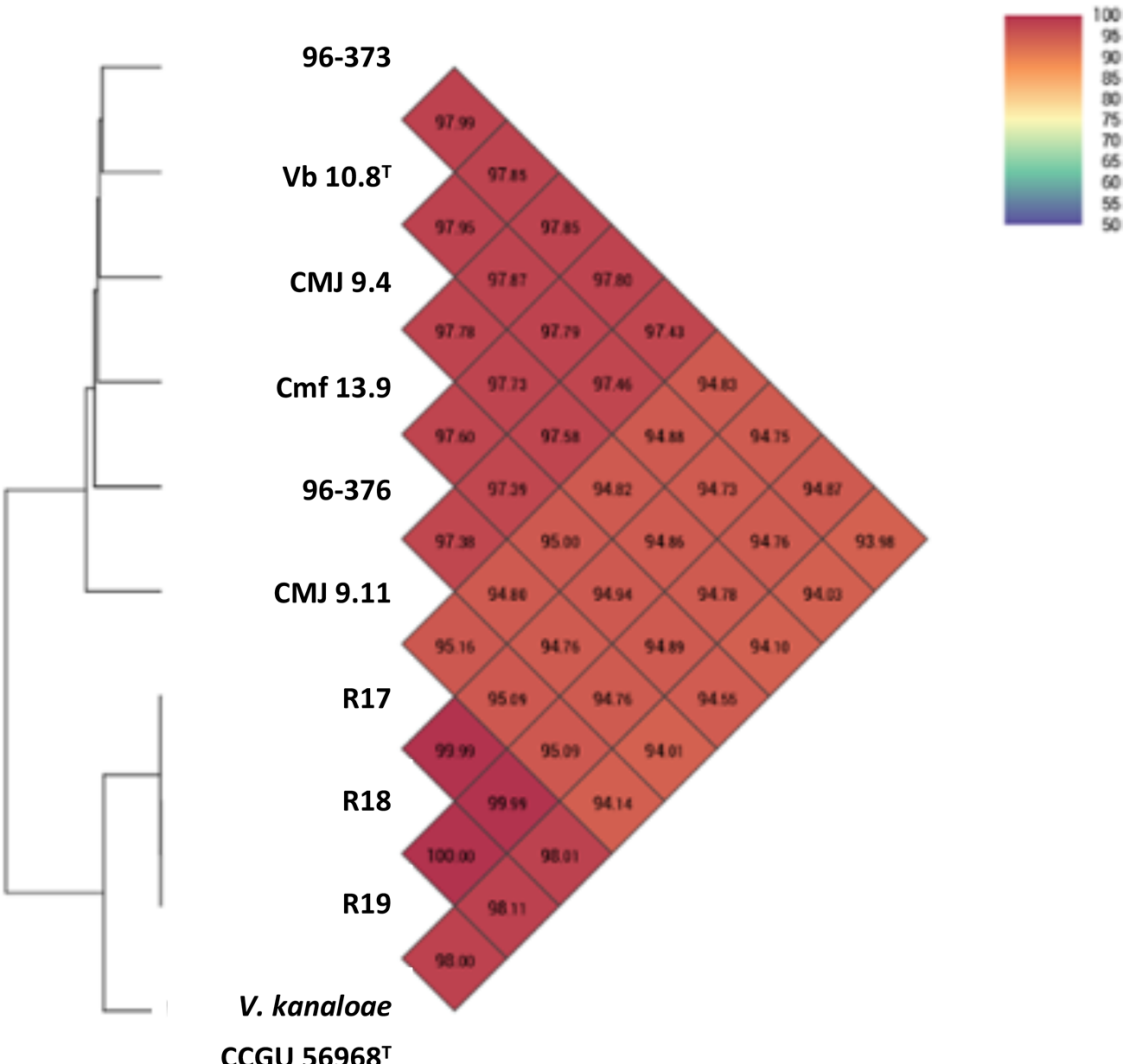
Values of OrthoANI for the strains of study

**Fig. 2:**
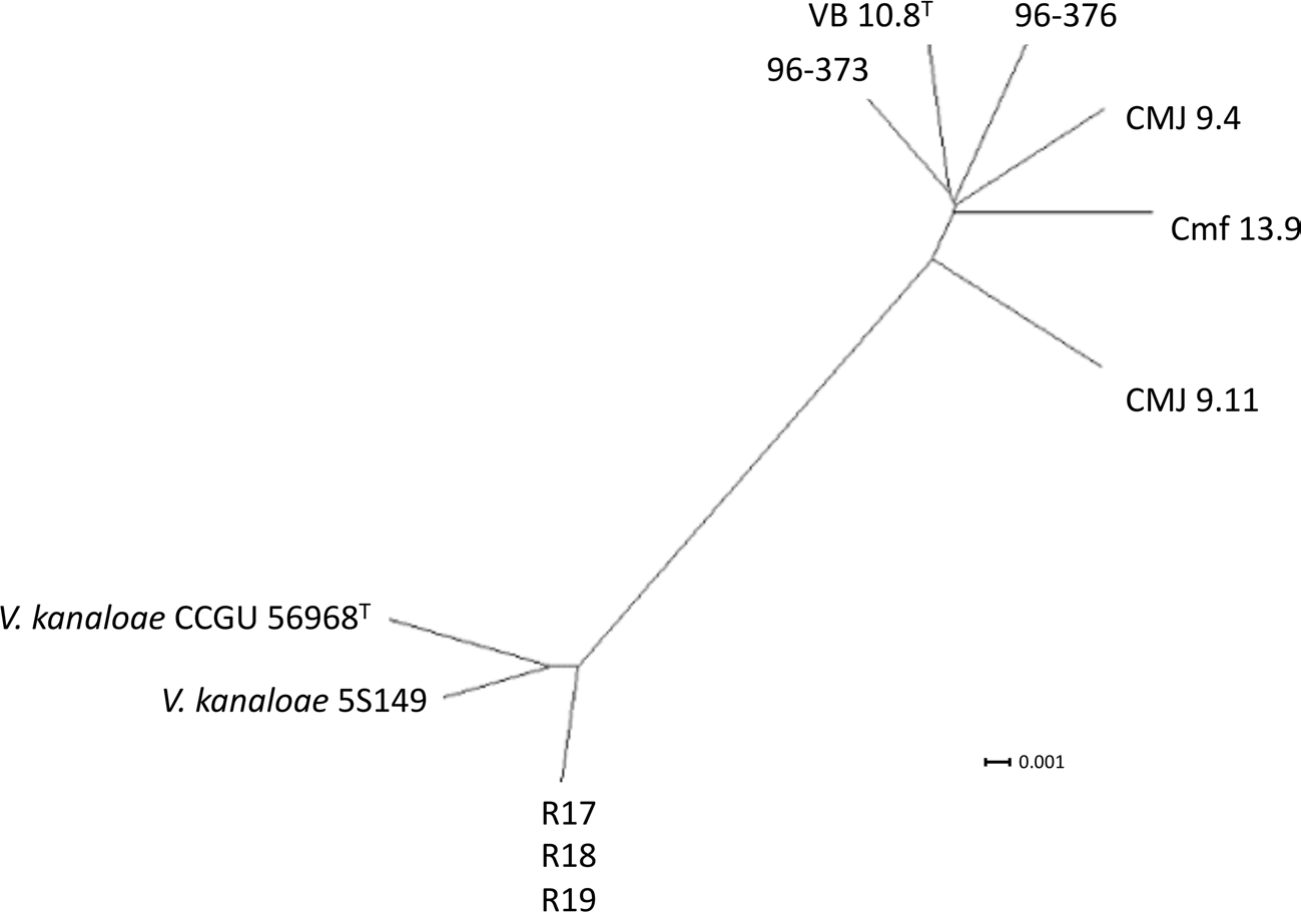
Phylogenetic tree of the core genome of *V. toranzoniae* and *V. kanaloae* strains

According to genome sequence similarity and genomic indices, the genomes of *V. toranzoniae* strains were separated into two well-defined clusters: one with six strains isolated from clams and seawater in Europe and the other with three strains isolated in Chile together with *V. kanaloae* (strains CCUG 56968^ T^ and 5S149)(Table 2; Figs. 1 and 2), a *Vibrio* species that was first isolated from diseased oyster (*Ostrea edulis*) larvae in France (Thompson et al. 2003). Our results also confirmed that the three Chilean isolates were clones, with a dDDH value of 100%, and an OrthoANI value of 99.99–100% (Table 2, Fig. 1). In addition, the OrthoANI and dDDH results showed that the Chilean isolates were *V. kanaloae*. OrthoANI and dDDH values between these isolates and *V. toranzoniae* strains were below the cutoff values proposed for the delineation of new species (< 96% and < 70%, respectively) (Konstantinidis and Tiedje 2005; Goris et al. 2007). In contrast, the values for these genomic indices were greater than 98.0% and 86%, with the type strain *V. kanaloae* CCUG 56968^ T^. Accordingly, the core-genome-based phylogenetic tree (Fig. 2) reinforced the existence of two separate monophyletic branches. Thus, based on these results, we proposed the assignment of Chilean isolates to *V. kanaloae*.

**Table 2:** Values of DDH among *V. toranzoniae* strains

	Vb 10.8^ T^	CMJ 9.4	CMJ 9.11	Cmf 13.9	96–373	96–376	R17	R18	R19	*V. kanaloae* CCUG 56968^ T^
Vb 10.8^ T^	100.0									
CMJ 9.4	81.2	100.0								
CMJ 9.11	77.9	78.5	100.0							
Cmf 13.9	80.7	79.6	77.1	100.0						
96–373	82.3	80.9	77.5	80.30	100.0					
96–376	80.6	80.4	77.1	78.90	80.00	100.0				
R17	58.7	58.9	60.6	59.5	58.6	58.6	100.0			
R18	58.5	58.9	60.6	59.4	58.5	58.5	100.0	100.0		
R19	58.5	58.9	60.6	59.4	58.5	58.5	100.0	100.0	100.0	
*V. kanaloae* CCUG 56968^ T^	61.6	62.0	61.0	62.5	61.8	61.8	86.4	86.3	86.3	100.0

After reviewing the genetic sequences available at NCBI, we discovered that one of the two sequences deposited as the 16S rRNA gene of the *V. kanaloae* type strain LMG 20539^ T^ was poorly named. Therefore, the 16S rRNA gene sequence with accession number AJ316193 (Thompson et al. 2001) coincided with *V. kanaloae* with 100% of similarity, followed by *V. toranzoniae* with 99.66%. Conversely, the other 16S rRNA gene sequence available, with accession number AM162657 (deposited by Le Chevalier et al., unpublished), corresponded to *V. atlanticus* (99.93% of similarity), followed by *V. tasmaniensis* (99.86%), *V. lentus* (99.78%), and then *V. toranzoniae* (98.78%) and *V. kanaloae* (98.77%).

The wrong sequence AM162657 was deposited in 2005, when the second most similar species, *V. tasmaniensis,* had already been described (Thompson et al. 2003). In addition, an identical sequence to AM162657 was submitted in 2011, with accession number NR_042468 and processed by NCBI staff, when both *V. tasmaniensis* and *V. atlanticus* 16S rRNA gene sequences were available. For the latter, the 16S rRNA gene sequence was deposited in 2007, with accession number EF599163 (Beaz-Hidalgo et al. 2008). This last mislabeled sequence (AM162657) was uploaded by the National Center for Biotechnology Information for its NCBI RefSeq Targeted Loci Project, which includes curated RefSeq records and selected validated GenBank sequences for curated BLAST databases.

It has been highlighted previously that sequences wrongly deposited as type strains may lead to errors in further studies that depend on public databases. This was the case for the so-called *Lelliottia nimipressuralis* type strain SGAir0187 (Heinle et al. 2018), that was misclassified due to a false type strain and was not a strain of the species (Salvà-Serra et al., 2019). Additionally, Beaz-Hidalgo and coworkers (2015) detected at least 12 misidentified *Aeromonas* genomes among the 44 that were deposited at the NCBI, insisting these authors are in the need of measures to prevent this kind of chaining errors.

In our case, the deposit of poor sequences led to the misassociation of the Chilean isolates with *V. toranzoniae* rather than *V. kanaloae* (Lasa et al. 2015). Considering all the results together and to avoid future problems, we have updated the taxonomic assignation of strain R17 and its deposited sequence (accession number GCA-001995825.2) to *V. kanaloae*.

### Genomic indices

The genome size of the *V. toranzoniae* strains studied ranged from 4.3 to 4.7 Mb, with 4.5 Mb being the average size of the species (Table 3). This genome size is in accordance with what was expected for a species of the *Vibrio* genus (Thompson et al. 2009). A minimum of 3826 and a maximum of 5184 coding sequences were predicted using the RAST annotation server for the different strains. For number of RNA genes oscillated between 126 and 188.

**Table 3:** Genome statistics for *V. toranzoniae* strains

	VB 10.8^ T^	CMJ 9.4	CMJ 9.11	Cmf 13.9	96–373	96–376
Genome size (Mb)	4.61	4.76	4.56	4.71	4.64	4.64
G + C content	44	43.9	43.8	43.9	43.9	43.9
Number of contigs	2	299	311	192	132	135
Coding sequences	4164	4236	4053	4158	4258	3826
RNA genes	172	158	181	181	126	146

The G + C content was practically the same among the strains, varying from 43.8 to 44 mol%, in the range for *Vibrio* species (Table 3). The OrthoANI values among *V. toranzoniae* isolates ranged from 94.73 to 100% (Fig. 1), and the dDDH values were between 58.50 and 100% (Table 2).

Although several studies have reported that genome size and G + C content are correlated with the ecological strategies of marine bacteria (Giovannoni et al. 2014; Luo and Moran 2015), our results did not reveal differences between the free-living bacteria (*V. toranzoniae* 96–373 and 96–376) and those associated with a host (*V. toranzoniae* Vb 10.8^ T^, CMJ 9.4, CMJ 9.11, Cmf 13.9).

### Complete genome sequencing of type strain *Vibrio toranzoniae* Vb 10.8^ T^

*V. toranzoniae* was first described based on four isolates from cultured clams in Galicia (NW Spain), designating the strain Vb 10.8^ T^ (= CECT 7225^ T^) as the type strain of the species (Lasa et al. 2013, 2016). For this strain, the read depth obtained by PacBio sequencing technology was 92 × ; the calculated genome size was 4,605,941 bp in length; and it was assembled in two contigs, which is consistent with the possession of two chromosomes by many species of the *Vibrio* genus, one larger and one smaller of approximately 3.2 and 1.4 Mb, respectively. The G + C content was 44 mol% and no plasmids were identified.

Complementation of short Illumina and long PacBio reads did not significantly improve the genome assembly, since the corrected genome size was 4,605,997 bp, which was only 56 bp longer than the length of the Pac-Bio-only sequenced genome.

With respect to strain 96–376, we were unable to close the genome, and complementation between Illumina and PacBio reads yielded six contigs with a total genome size of 4,370,366 bp, that is, 31,016 bp less than that of the PacBio-only assembly.

### Core- and pangenome analysis of *V. toranzoniae*

**Fig. 3:**
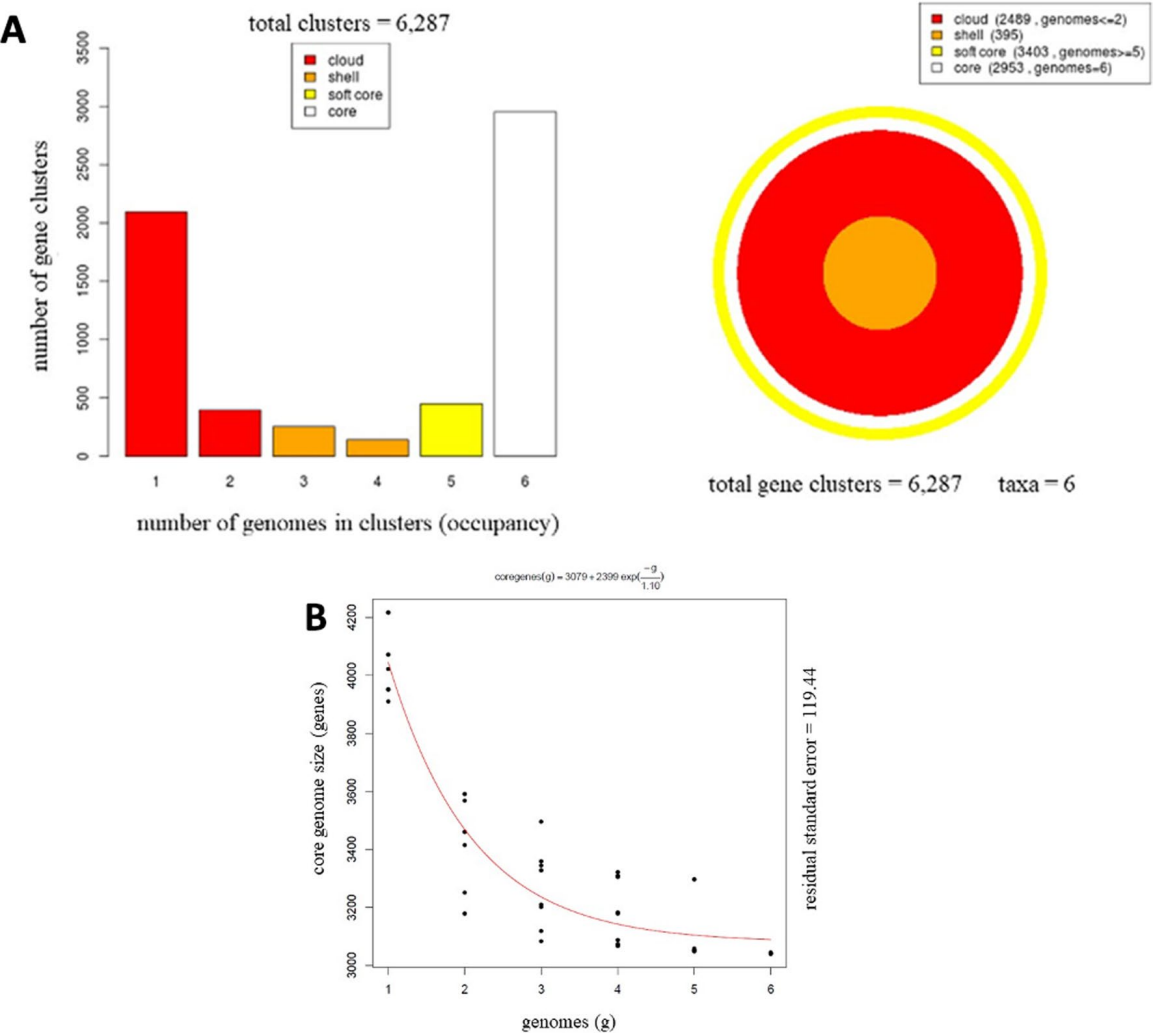
Pangenome analysis of *V. toranzoniae* strains. **A** Partition of the OMCL pangenomic matrix into shell, cloud, soft-core, and core compartments. **B** Estimate size of core genome

**Fig. 4:**
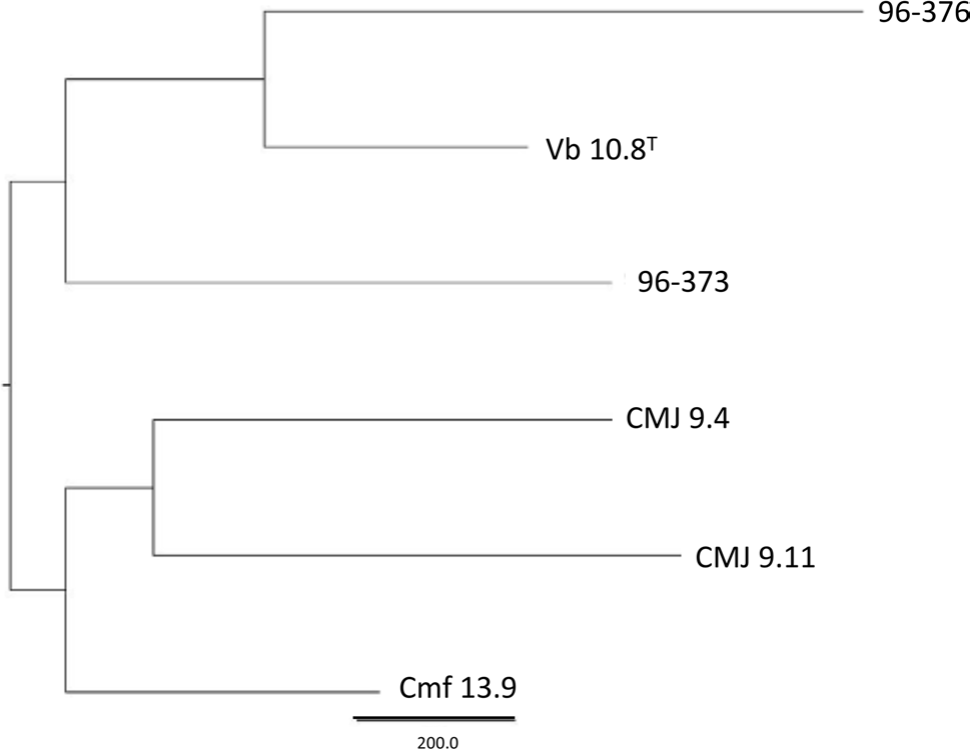
Phylogenomic tree based on pangenomic matrix of *V. toranzoniae* strains

Genome analysis of the six *V. toranzoniae* isolates included in the study with GET_Homologues revealed a pangenome of 6287 gene clusters. Of these 6287 genes, 2489 genes were only present in only 2 or fewer taxa (cloud genome), 395 genes were shared by 3 or 4 taxa (shell genome), 3404 were shared by 5 isolates or more (soft core), and 2953 genes were showed by all strains studied (core genome) (Fig. 3A). As shown in Fig. 3B, the core genome moderately decreased when more genomes were included, while the pangenome exhibited the opposite trend. A phylogenomic tree based on the pangenomic matrix of *V. toranzoniae* strains is shown in Fig. 4. Using Roary software, from the total pangenome of 6132 genes, 2766 genes were found to belong to the core genome (shared by 99–100% of taxa), whereas 3366 genes formed the shell genome (shared by 15–95% of taxa).

Moreover, the phylogenomic analysis of the core genome (Fig. 2) did not reveal a differentiation between strains according to the lifestyle (commensal or free-living) either. However, when examining the phylogenomic tree (Fig. 4), we observed the clustering of the three strains isolated from clams in Camariñas (Galicia, Spain), thus sharing the same growing area. Since the pangenome comprises more genes, including those not shared by all strains, this could indicate a local episode of horizontal gene transfer. Consequently, geographical conditions appear to be more decisive than lifestyle or host in *V. toranzoniae* strains. Further studies are needed to confirm such hypothesis.

### Genomic features

Although phylogenetic divergence was not observed between strains with different lifestyles, some notable differences in gene content were observed.

All the strains except one, the environmental strain 96–376, presented genes related to flagellar synthesis and regulation. The absence of motility in the 96–376 isolate was similarly observed in soft agar and checked by optical microscopy. The remaining strains exhibited motility in both soft agar and optical microscopy; flagella were stained and observed in bacterial preparations via 100 × optical microscopy (data not shown).

Likewise, the environmental strain 96–376, together with the other strain isolated from seawater 96–373, did not exhibit the genes for the rhamnose synthesis pathway, involved in the synthesis of the capsule. This biosynthetic pathway is common and highly conserved across both Gram-positive and Gram-negative bacteria, and involves four distinct enzymes that transform glucose into dTDP-L-rhamnose. The initial enzyme in this pathway, glucose-1-phosphate thymidylyltransferase, is responsible for attaching a thymidylmonophosphate nucleotide to Glu-1-P. The resulting dTDP-glucose is further oxidized and dehydrated by the enzyme dTDP-d-glucose 4,6-dehydratase. Subsequently, a third enzyme, dTDP-6-deoxy-d-xylo-4-hexulose 3,5-epimerase, facilitates double epimerization at the C3 and C5 positions. In the final step, the dTDP-6-deoxy-l-lyxo-4-hexulose reductase reduces the C4 keto group to produce the final product, dTDP-l-rhamnose. On the other hand, the type strain Vb 10.8^ T^ lacked the reductase gene in the dTDP-rhamnose pathway, and the strain Cmf 13.9 was the only isolate hosting the thymidylyltransferase.

The presence of capsules was also assessed by growth on CRA plates. After 48 h of incubation, all the strains presented black colonies indicating the production of capsules, although according to the absence of rhamnose-synthesis pathway, strains 96–373 and 96–376 had the lowest production, indicating that rhamnose is important but not exclusive for capsule production. The presence of capsules in all the strains could be explained by the advantages that the extracellular polysaccharides confer not only for environmental survival, but also for host invasion, colonization, persistence, and eventually pathogenesis (Bian et al. 2021). Contrary to what it was initially thought, capsules provide protection from physical and chemical stresses without the detriment of a high transfer of genetic materials between bacteria (Rendueles et al. 2018).

All the strains presented genes for the transport of iron and for the siderophore aerobactin, although the aerobactin synthase protein IucC was present only in strain 96–373. Nevertheless, only the strain Cmf 13.9 contained a kit of genes for the siderophore assembly. Accordingly, Cmf 13.9 was the only isolate capable of forming an orange halo around blue around the colonies on CAS plates, which is indicative of siderophore production.

Related to virulence factors (Table 4), all the strains hosted a vibriolysin and a hemolysin (putative for the case of CMJ 9.11). In addition, all the strains exhibited T1SS-secreted agglutinin repeat-in-toxins (RTX), with the exception of the type strain Vb 10.8^ T^. The strains also exhibited the presence of the related Ca^2+^ binding proteins, the type I secretion system, and components necessary for the extracellular secretion, such as the TolC outer membrane protein, an ATP-binding cassette (ABC), and a LapC membrane fusion protein. Despite the presence of vibriolysins and hemolysins, which have been described as virulence factors in other *Vibrio* species(Yuan et al. 2020; Galvis et al. 2021), none of the *V. toranzoniae* strains cause mortality in clams or turbot (data not shown). This led us to speculate that vibriolysins might not be expressed or that some of the regulatory factors are absent. These observations suggest that the *V. kanaloae* strain R17 (reclassified in this work) isolated from moribund red conger eel in Chile could have been the responsible etiological agent; thus, *V. toranzoniae* would remain only as a potential pathogen.

**Table 4:** Summary of genetic traits present in *V. toranzonaie* strains

	CRISPR sequences	Incomplete prophage sequences	Secondary metabolites	Virulence factors	Phage defense elemente
Vb 10.8^ T^	1	1	PUFAs, ectoine, arylpolyene, bacteriocine, betalactone	Hemolysin, VirK, virulence-associated E family protein, iron-regulated protein IrgB	RM (3), Druantia, Zorya, Cas, dGTPase, Viperin
CMJ 9.4	3	2	PUFAs, ectoine, arylpolyene, bacteriocine, betalactone	Hemolysin, iron-regulated protein IrgB	RM (2), Cas (2), dGTPase, BstA
CMJ 9.11	1	3	PUFAs, ectoine, arylpolyene, bacteriocine, betalactone	Hemolysin, VirK	RM (2), dGTPase, BREX, DTR, Cas, Rst-sirtuin-like
Cmf 13.9	2	1	PUFAs, ectoine, arylpolyene, bacteriocine, betalactone, siderophore	Hemolysin, probable RTX, iron-regulated protein IrgB, siderophore assembly kit	RM (2), dGTPase, Cas, Rst-sirtuin-like
96–373	1	1	PUFAs, ectoine, arylpolyene, bacteriocine, betalactone, siderophore	Hemolysin, iron-regulated protein IrgB	RM (3), dGTPase, Septu, Hachiman
96–376	1	0	PUFAs, ectoine, arylpolyene, bacteriocine, betalactone	Hemolysin, iron-regulated protein IrgB	RM (2), Nhi, Zorya, Kiwa, dGTPase, Rst-ATPase

All strains harbor the antimicrobial peptides adeF, CRP, QnrS2, drfA6

Genomic differences between closely related strains are usually concentrated in strain-specific regions of chromosomes known as genomic islands; these regions are generally acquired by HGT and that contain adaptive traits that can be linked to niche adaptation (Dobrindt et al. 2004, Penn et al. 2009). Using IslandViewer 4, Genomic Islands (GIs) were identified by SIGI-HMM and Island-Path-DIMOB methods, but not by the IslandPick method (Table 5). For all the strains, the highest number of GIs was found by the SIGI-HMM method. The strain with the highest GI number was CMJ 9.4. Among the identified GI, mobile elements, phage proteins, glycosyltransferases, lipid metabolism proteins, and hypothetical proteins were the most common proteins. Iron acquisition system proteins, L-ectoine synthase, and MSHA pilin proteins were also found.

**Table 5:** Number of identified GIs in *V. toranzoniae* strains

	Vb 10.8^ T^			CMJ 9.4			CMJ 9.11			Cmf 13.9			96–373			96–376		
	S	I	T	S	I	T	S	I	T	S	I	T	S	I	T	S	I	T
*V. anguillarum*	29	8	37	33	12	45	25	13	38	26	12	38	29	10	39	26	12	38
*V. splendidus*	27	9	36	34	10	44	28	15	43	26	9	35	30	12	42	26	10	36
*V. vulnificus*	30	9	39	34	9	43	26	14	40	25	10	35	28	12	40	27	10	37

*S*, SIGI-HMM method, *I*, IslandPath-DIMOB method, *T*, total

### Horizontal gene transfer evidences

The evidenced high gene transfer was assessed by different indicators. For example, the abundance of secondary metabolites is indicative of genomic exchange since many of these metabolites are acquired by horizontal gene transfer (Khaldi et al. 2008). A total of six secondary metabolites were identified using AntiSMASH (Table 4). Among them, five were distributed in all the strains (polyunsaturated fatty-acid (PUFA) cluster, ectoine, bacteriocin, arylpolyene, and betalactone). These secondary metabolites are related to the adaptation of the bacteria to marine environments (Jensen and Fenical 1996, de Carvalho and Fernandes 2010) and are found in different marine bacterial genera. Thus, PUFAs are produced by different marine bacteria such as *Vibrio*, *Photobacterium*, *Psychromonas*, and *Shewanella*, enabling the transportation of nutrients through the membrane and maintaining its fluidity in the deep-sea cold environment inhabited by these genera (Moi et al. 2018). Aryl polyenes are natural bacterial products that protect bacteria from reactive oxygen species (Schöner et al. 2016), whereas bacteriocin and betalactone are compounds produced by bacteria that show inhibitory or killing effects on other cells (Manivasagan et al. 2014; Yang et al. 2014). Additionally, ectoine is an organic compound whose accumulation within the cell allows bacteria to maintain turgor pressure under high osmolarity, thus contributing to cell resistance against saline stress (Gregory et al. 2019). Here, we found genes coding for ectoine synthesis in genomic islands that are usually enriched in secondary metabolites genes, providing evidence that secondary metabolism is linked to functional adaptation (Penn 2009). Finally, a siderophore cluster was recognized in only two strains, namely Cmf 13.9 and 96–373, consistent with what we observed in the genome browser.

The variable possession of antiphage systems in closely related strains, as in our case, indicates a high rate of horizontal gene transfer (Tesson et al. 2022). These systems were identified for all the strains (Table 4), on a number ranging from five to eight, as the average number for prokaryotic genomes is five (Tesson et al. 2022). Among them, all the strains encode for RM and dGTPase, the most common antiphage systems together with Cas which, interestingly, is only present in the strains isolated from clams.

All the strains presented CRISPR sequences varying from 1 to 3 (Table 4). For the case of Cas cluster gene sequences, only the strains CMJ 9.4 and CMJ 9.11 presented 2 and 1, respectively. No intact prophage sequence was detected, although the majority of the strains had 1 to 3 incomplete prophage sequences (Table 4). Only the environmental strain 96–376 did not present any prophage sequence, neither intact nor incomplete nor questionable.

Using CARD, four antibiotic resistance gene sequences were identified for all the strains (Table 4), coding for the quinolone resistance protein QnrS2, two resistance-nodulation-cell division antibiotic efflux pumps (adeF and CRP), and the trimethoprim-resistant dihydrofolate reductase dfrA6.

## Conclusions

The comparative genomic analysis of the *V. toranzoniae* strains revealed ample homology between them, with differences related to motility, capsule synthesis, the iron acquisition system, and phage-related elements. The strains shared a core genome of 2953 genes out of a pangenome of 6287 genes, according to GET_Homologues. Those strains grown in the same clam breeding area were grouped phylogenetically together; thus, the geographical conditions prevailed over the ecological conditions. Finally, reclassification of the R17 strain as *V. kanaloae* emphasizes the need for deposited sequences to be cured and properly designated to avoid possible mistakes, especially among strains as similar as those belonging to the Splendidus clade within the genus *Vibrio*.

## Data Availability

Sequence data that support the findings of this study have been deposited in GenBank under the accession numbers GCA-001541335.1 (*V. toranzoniae* CECT 7225 T), GCA-009906155.1 (*V. toranzoniae* 96–373), GCA-009906235.1 (*V. toranzoniae* 96–376), GCA-009906185.1 (*V. toranzoniae* CMJ 9.4), GCA-009906175.1 (*V. toranzoniae* CMJ 9.11), GCA-009906085.1 (*V. toranzoniae* Cmf 13.9), and GCA-001995825.2 (*V. kanaloae* R17).
